# Parental information about the option to apply for pregnancy termination after the detection of a congenital abnormality and factors influencing parental decision-making: a cohort study

**DOI:** 10.1186/s12884-022-05255-0

**Published:** 2022-12-17

**Authors:** Karina Hjort-Pedersen, Annette Wind Olesen, Ester Garne, Pernille Mathiesen Toerring, Chunsen Wu, Lene Sperling

**Affiliations:** 1 Research Unit of Gynaecology and Obstetrics, Odense University Hospital, University of Southern Denmark, Odense, Denmark; 2 Odense Patient Data Explorative Network, OPEN, Odense University Hospital, Odense, Denmark; 3 Department of Clinical Research, University of Southern Denmark, Odense, Denmark; 4 Department of Gynaecology and Obstetrics, Odense University Hospital, Odense, Denmark; 5 Department of Paediatrics and Adolescent Medicine, Lillebaelt Hospital, University Hospital of Southern Denmark, Kolding, Denmark; 6 Department of Clinical Genetics, Odense University Hospital, Odense, Denmark

**Keywords:** Prenatal screening, Ultrasound, Congenital abnormalities, Termination of pregnancy, Parental decision

## Abstract

**Background:**

The detection of an abnormality during prenatal screening implies that the parents are informed about possible treatment and management of the pregnancy, birth, and postnatal course. This information should enable the parents to make decisions regarding the pregnancy, especially in cases where termination of pregnancy may be an option. The objectives of this study were to investigate how often doctors informed parents about pregnancy termination when the fetus had an anomaly and which demographic factors were related to parental decision-making.

**Methods:**

This was a retrospective cohort study with prospectively collected data of fetuses diagnosed with an abnormality during prenatal screening between 2014 and 2016 in Denmark. We categorized the abnormalities into five long-term prognosis groups and analyzed their association with the doctor provided information about termination. We tested the association between demographic variables and parental decisions using univariate and multivariate statistical analyses.

**Results:**

Three hundred and twenty fetuses were diagnosed with an abnormality. In 67% of these cases, the parents were informed about termination. All parents whose fetus had a lethal prognosis were informed about termination. By comparison, the parents of 98% of fetuses with genetic disorders, 96% of fetuses with poor prognosis, 69% of fetuses with uncertain prognosis, and 12% of fetuses with good prognosis were informed about termination. Of these parents, 92% chose to terminate. A lethal long-term prognosis was the only factor related to parental decision to terminate a pregnancy.

**Conclusions:**

Doctors mainly informed parents about the option of pregnancy termination for conditions with a poor or lethal long-term prognosis or for genetic disorders. Only conditions with a lethal prognosis were significantly related to the parental decision to terminate the pregnancy.

**Supplementary Information:**

The online version contains supplementary material available at 10.1186/s12884-022-05255-0.

## Background

Prenatal screening aims to gain knowledge about the fetus. If the result of the examinations is abnormal, the parents are informed about possible treatment and management of the pregnancy, birth, and postnatal course. This information should enable parents to make decisions regarding pregnancy, especially in cases where termination of pregnancy may be an option [1]. The use of new genetic testing methods (microarray) improved ultrasound equipment in prenatal diagnostics, and better education of sonographers and doctors has led to higher prenatal detection rates earlier in gestation. Furthermore, it has enabled the diagnosis of diseases that were previously undetectable prenatally, including diseases of a less severe nature [2]. The prevalence of termination of pregnancy due to malformations or genetic disorders varies in Europe according to prenatal screening policy and abortion legislation [3].

The wording of abortion legislation in different countries often leaves room for interpretation and has been reported to be a challenge [4]. Studies have shown that medical professionals think it is difficult to define terms such as “substantial” and “serious” in the legislation [5]. The estimation of how severe a condition has to be to justify termination is influenced by views on impairment and disability among parents, medical professionals, and society [5, 6]. The Danish abortion legislation allows free abortion up till 11 + 6 weeks of gestation. From week 12 + 0, termination of pregnancy must be approved by a regional abortion council. An additional file describes the Danish abortion legislation in more detail (see Additional file 1). In Denmark, the most severe cases often result in the termination of pregnancy in the first and second trimesters before the fetus becomes viable [7, 8]. In our study, counseling was performed with specialized pediatricians, surgeons, and/or geneticists. It is usually the specialists in fetal medicine/obstetricians who decide in which cases information about termination is given. The Danish prenatal screening program consist of a first trimester and second trimester scan and is described in more detail in an additional file (see Additional file 2).

The aims of this study were to investigate how often doctors informed parents about pregnancy termination when the fetus had an anomaly and which demographic factors were related to parental decision-making.

## Methods

### Study design and population

We conducted a retrospective cohort study with prospectively collected data from fetuses alive at the first-trimester scan (FTS) and the second-trimester scan.

The study included four obstetric departments in a region of Denmark between February 2014 and September 2016. The hospitals include one tertiary referral center and three community hospitals.

The study population included all fetuses with a malformation and/or genetic disorder detected between gestational weeks 12 + 0 and 22 + 6.

We stratified abnormalities into two main categories: (1) Fetuses with genetic disorders with or without ultrasound verified malformation and (2) Fetuses with malformations without prenatally diagnosed genetic disorders (Fig. 2).

We classified the validated abnormalities into five groups according to their long-term prognosis, as this has been described as being the primary focus of parents when making the decision [9–11]. We classified cases with more than one malformation in the same organ system according to the most severe diagnosis and cases with malformations in two or more organ systems as multiple malformations:

1. Conditions with good long-term prognosis: Isolated malformations with no suspicion of genetic disorders and the possibility for optimal treatment that may cure the condition or without the need for treatment.
2. Conditions with uncertain long-term prognosis: Isolated malformations without genetic disorders and possible/available treatment that may cure the condition but without certainty, or with a higher risk of death or disability.
3. Conditions with poor long-term prognosis: Malformations without genetic disorders. Incurable conditions where treatment ensures the infant’s survival and stabilizes the condition, but with a prospect of severe permanent physical or mental disability.
4. Conditions with a lethal prognosis: Conditions that are incompatible with life.
5. Conditions with pathogenic genetic disorders: This category includes lethal and nonlethal genetic disorders.

Each diagnosis was discussed among the authors. We did not have information on the severity of each case, the number of possible surgeries or hospitalizations, making the classification more general. Information about pregnancy termination were recorded for each abnormality.

The study’s main outcome was an estimation of whether parents/women were informed about termination after detection of a malformed fetus, according to the long-term prognosis of the malformation. Furthermore, the parental decision to terminate or continue the pregnancy was made according to the long-term prognosis of the malformation. Finally, demographic variables related to pregnancy termination after information was determined. We divided the group that received information about pregnancy termination into one group that opted for termination and another group that wished to continue the pregnancy.

Gestational age at diagnosis was categorized into first trimester (gestational age 12 + 0 to 15 + 6) and second trimester (gestational age 16 + 0 to 22 + 6). BMI groups were initially categorized according to the World Health Organization (WHO) definition [12] (< 18.5, 18.5–24.9, 25.0–29.9, ≥ 30.0), but due to the limited number of women under 18.5, we combined the first two BMI categories into a single category. We used a cubic spline to explore potential categorization for age (18–24, 25–29, 30–34, ≥ 35). Due to small numbers in the ethnicity variable, we combined Asian, Hispanic, and African American as a single category.

### Data collection

We extracted data from the ultrasound database Astraia (Astraia software gmbh, version 1.24.7, Germany, https://www.astraia.com/en/), patient administrative systems (PAS), and medical records in each hospital. All Danish residents have a unique civil registration number (CRN) enabling cross-referencing between registers. Eligible validated cases and data were registered in a Research Electronic Data Capture (REDCap) database [13, 14].

**Fig. 1:**
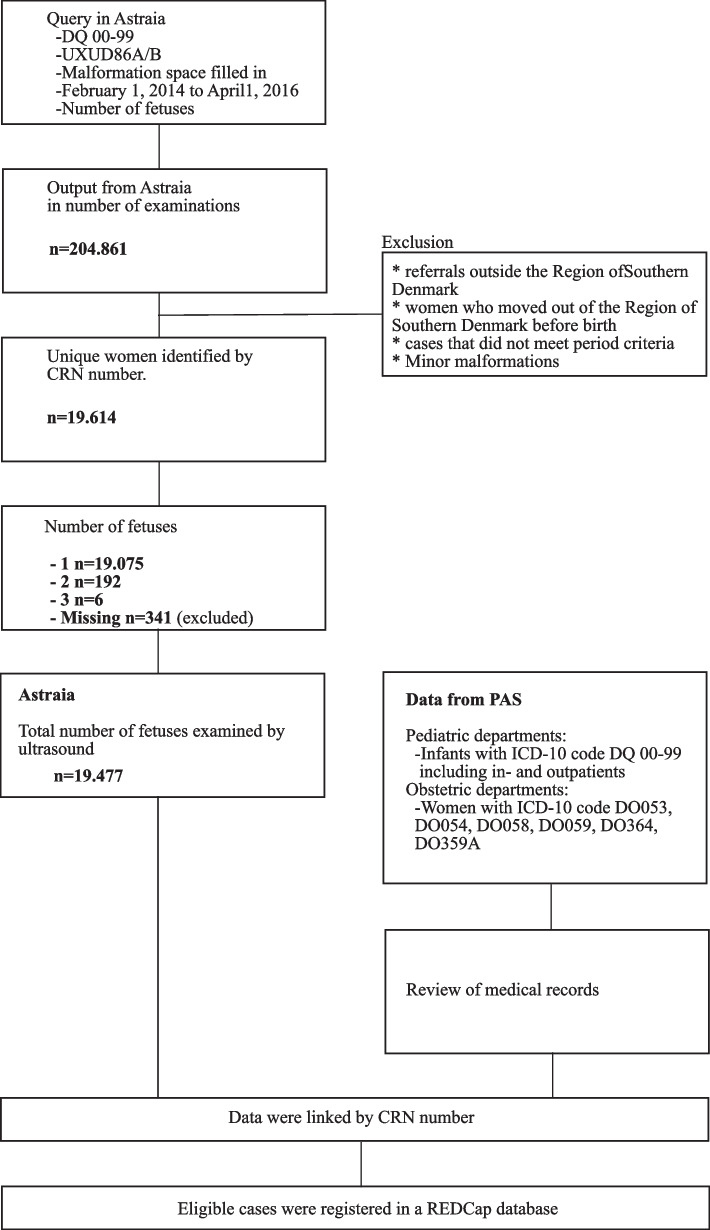
Flowchart of the selection of the study population

We identified all fetuses with either procedural code UXUD86A/B, ICD-10 diagnosis code DQ 00–99, or with data on malformations or genetic disorders that were entered in the organ-specific tick-boxes. Unique cases were identified by the pregnant woman’s unique civil registration number (CRN), which is given to all Danish citizens at birth. The number of fetuses for each woman was registered in Astraia and was used to create the study population. We excluded cases if the woman was referred from outside the Region of Southern Denmark, the woman moved out of the region before giving birth, the malformations were minor or undetectable by ultrasound (e.g., hypospadias and small muscular ventricular septal defects (VSD)), and if the number of fetuses was missing (Fig. 1).

Data on eligible caseswas obtained from PAS and were identified by reviewing medical records. Demographic and clinical variables from Astraia included estimated date of delivery, maternal age (continuous), body mass index (BMI) (continuous), smoking status (yes/no), civil status (single/cohabiting), parity (nulliparous/multiparous), type of conception (spontaneous/assisted), number of fetuses (singleton/twins), gestational age at diagnosis (weeks) and ethnicity (Caucasian/Asian/Hispanic/Black).. Data of the validated cases were registered in a REDCap database.

Data from the pediatric departments included data on live-born infants born during the study period with malformation or genetic diagnosis corresponding to ICD-10 code DQ 00–99 within six months after birth. Data included in- and outpatients. We reviewed medical records for all the cases to identify eligible cases and validate them. Cases were identified by the infant’s CRN and linked with their mother’s CRN in Astraia. Data from the obstetric departments included data on women who gave birth or terminated a pregnancy due to a malformation or genetic disorder in the study period corresponding to ICD-10 code DO053, DO054, DO058, DO059, DO364 DO359A. All cases were reviewed in the medical records to validate and identify eligible cases. Cases were identified by the women’s CRN and linked with data from Astraia. Only prenatally detected cases were included (Fig. 1).

We used medical records and autopsy reports to validate the cases identified in Astraia and PAS. Furthermore, we obtained data regarding the termination of pregnancy, including the information given by health care professionals on the of applying for termination. Data on gestational age when parents were informed and their decision to continue or terminate the pregnancy were also obtained. Validation was performed with two specialists in fetal medicine and a pediatric consultant.

### Statistical analysis

All categorical data are presented as numbers and percentages according to parents’ decisions (termination/continuation of pregnancy), and the chi-square test or Fisher’s exact test were used to test the distribution.

Univariate analysis for the association between demographic variables and the decision to terminate or continue the pregnancy was conducted. Given the frequent events in this study, we estimated the risk ratio (RR) for each risk factor.

The original plan was to include all clinically significant variables and statistically significant (*p* ≤ 0.05) variables in a multivariate regression analysis of the association with the termination of pregnancy. Even though the number of cases (termination of pregnancy) was sufficient, the number of non-cases (continuation of pregnancy) was too low. Therefore, multivariate regression only included statistically significant variables from the univariate analysis, and numbers not too small.. Generalized linear regression with a log link and robust variance estimator was used to estimate RR with 95% confidence interval (CI). Stata 15 was used for statistical analysis.

### Ethical approval

All analysis were carried out in accordance with current Danish guidelines and regulations. The study was approved by the Danish Data Protection Agency (18/43849). Access to register-based health data and data from medical records were granted by the Danish Health Authority (3–3013-806/1/).

### Consent to participate

The Ethics Committee of Health Research Ethics for Southern Denmark (S-20142000HLP) waived the need for informed consent.

## Results

**Fig. 2:**
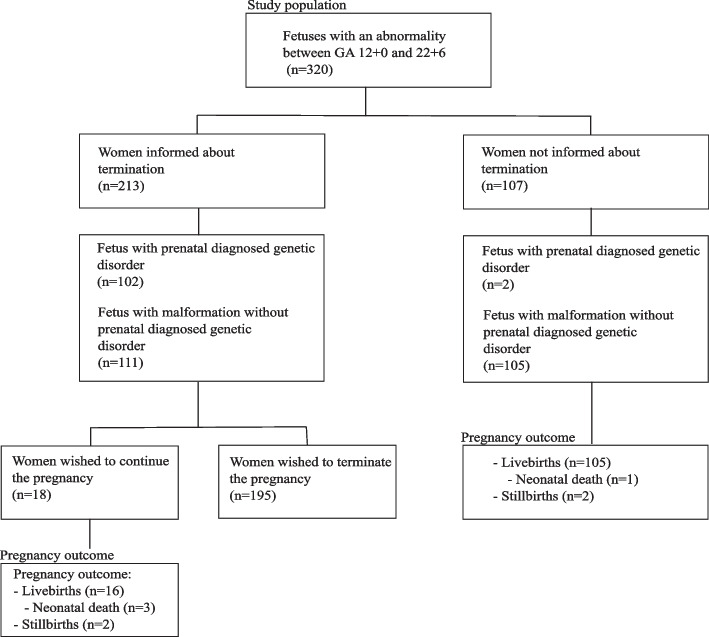
Study population

The study population is shown in Fig. 2.

We found 320 fetuses with prenatally detected abnormalities. The number of cases for each of the five prognosis groups is shown in Table 1. In total, 67% (n = 213) were informed about the termination of pregnancy as an option (Fig. 2). Information about termination according to each prognosis group is shown in Table 1. In cases with a lethal prognosis, all parents were informed about termination; in cases with genetic disorders, 98%; with a poor long-term prognosis, 96%; with uncertain long-term prognosis, 69%; and with a good long-term prognosis, 12% (Table 1).

**Table 1:** Parental information about termination according to five prognostic categories. The specific abnormalities are listed for each category

	Cases total		Information about termination			
Classification of fetal abnormalities according to prognosis	(n)	(%)	No (n)	(%)	Yes (n)	(%)
**1. Conditions with a good long-term prognosis**	**107**	**33.4**	**94**	**88**	**13**	**12**
Micrognathia			< 5		0	
Unilateral cleft lip			8		0	
Diaphragmatic hernia			< 5		< 5	
Coarctation of aorta			< 5		0	
Right aortic arch			< 5		0	
Persistent left superior vena cava			< 5		0	
Other malformation of the tricuspid valve			0		< 5	
Ventricular septal defect			< 5		0	
Double kidney			< 5		0	
Hydronephrosis			36		0	
Renal agenesis unilateral			< 5		0	
Cystic renal dysplasia unilateral			10		0	
Duplication of the renal pelvis			< 5		0	
Omphalocele			0		< 5	
Gastroschisis			6		< 5	
Intestinal atresia			< 5		< 5	
Anal/rectal atresia			< 5		0	
Clubfoot			11		0	
Congenital malformation of female genitalia			< 5		0	
**2. Conditions with uncertain long-term prognosis**	**26**	**8.1**	**8**	**31**	**18**	**69**
Cleft lip-palate/facial cleft bilateral			< 5		< 5	
Pulmonary adenoid malformation			< 5		0	
Transposition of the great arteries			0		< 5	
Common arterial trunk			0		< 5	
Other malformation of aorta			0		< 5	
Tetralogy of Fallot			< 5		< 5	
Amelia of the lower limb			0		< 5	
Pelvic teratoma			0		< 5	
Lymphangioma			0		< 5	
Hygroma/hydrops			0		8	
Short long bones			< 5		0	
Small biometries			< 5		< 5	
**3. Conditions with poor long-term prognosis**	**70**	**21.9**	**< 5**	**N/A**	**67**	**N/A**
Spina bifida			0		13	
Cerebellar hypoplasia			< 5		< 5	
Agenesis of corpus callosum			0		< 5	
Occipital encephalocele			0		< 5	
Congenital hydrocephalus			0		< 5	
Other specified congenital malformations of brain			0		< 5	
Hypoplastic left heart syndrome			0		9	
Hypoplastic right heart syndrome			0		7	
Ebsteins anomaly			0		< 5	
Congenital cardiomyopathy			0		< 5	
Cystic renal dysplasia bilateral			0		< 5	
Autosomal recessive polycystic kidney disease			0		< 5	
Megacystis/urethral valves			0		< 5	
Multiple malformations			< 5		21	
**4. Conditions with a lethal prognosis**	**13**	**4.1**	**0**	**0**	**13**	**100**
Anencephaly			0		9	
Holoprosencephaly			0		< 5	
Renal agenesis bilateral			0		< 5	
**5. Conditions with genetic disorders**	**104**	**32.5**	**< 5**	**N/A**	**102**	**N/A**
Aneuploidy						
Trisomy 13			0		10	
Trisomy 18			0		21	
Trisomy 21			0		43	
Triploidy			0		< 5	
** Sex chromosomal abnormality**						
Klinefelter syndrome			< 5		< 5	
Triple X syndrome			0		< 5	
Turner syndrome			< 5		10	
Monogenic disorder			< 5		< 5	
**Copy number variation (pathogenic)**			0		10	
**Total**	**320**	**100**	**107**	**33**	**213**	**67**

Numbers are reported as < 5 to comply with data protection regulations

N/A: not applicable

The 107 cases where the parents did not receive information about termination mainly included conditions with a good long-term prognosis, such as clubfoot, hydronephrosis, unilateral cleft lip, gastroschisis, and unilateral renal dysplasia (Table 1). Of these cases, 6% (n = 6) were diagnosed with an additional genetic disorder after birth (four children with monogenic disorders, one with a copy number variation (CNV), and one with aneuploidy). The association between malformations and genetic disorders is shown in Additional file 3.

Of parents informed about pregnancy termination, 92% (n = 195) decided to apply for termination (Table 2).

**Table 2:** Parental decision after information about termination according to five prognostic categories. The specific abnormalities are listed for each category

Prognosis of the conditions	Parental decision			
	Wish to terminate the pregnancy		Wish to continue the pregnancy	
	N	%	N	%
**1. Conditions with a good long-term prognosis**	**9**	**N/A**	**< 5**	**N/A**
Diaphragmatic hernia				
Other malformation of tricuspid valve				
Omphalocele				
Gastroschisis				
Intestinal atresia				
**2. Conditions with uncertain long-term prognosis**	**12**	**67**	**6**	**33**
Cleft lip bilateral/ facial cleft bilateral				
Transposition of the great arteries				
Common arterial trunk				
Other malformations of aorta				
Tetralogy of Fallot				
Amelia of the lower limb				
Pelvic teratoma				
Lymphangioma				
Hygroma/hydrops				
Small biometries				
**3. Conditions with poor long-term prognosis**	**60**	**90**	**7**	**10**
Spina bifida				
Cerebellar hypoplasia				
Agenesis of corpus callosum				
Occipital encephalocele				
Congenital hydrocephalus				
Other specified congenital malformations of the brain				
Hypoplastic left heart syndrome				
Hypoplastic right heart syndrome				
Ebsteins anomaly				
Congenital cardiomyopathy				
Cystic renal dysplasia bilateral				
Autosomal recessive polycystic kidney disease				
Megacystis/urethral valves				
Multiple malformations				
**4. Conditions with a lethal prognosis**	**13**	**100**	**0**	**0**
Anencephaly				
Holoprosencephaly				
Renal agenesis bilateral				
**5. Conditions with a genetic disorder**	**101**	**N/A**	**< 5**	**N/A**
**Total**	**195**	**92**	**18**	**8**

Numbers are reported as < 5 to comply with data protection regulations

N/A: not applicable

The decision to terminate or continue the pregnancy was associated with singleton pregnancies, conditions diagnosed in the first trimester, conditions with a lethal prognosis, and conditions with a genetic disorder (Table 3).

**Table 3:** Demographic variables of women deciding to terminate or continue the pregnancy after information about termination

Demographic data	Total (n)	Total (%)	Termination of pregnancy (n)	(%)	Continuation of pregnancy (n)	(%)
**Total**	213	100.0	195	92.0	18	8.0
**Age group**						
18–24	24	11.3	22	11.3	2	11.1
25–29	61	28.6	54	27.7	7	38.9
30–34	61	28.6	56	28.7	5	27.8
≥ 35	67	31.5	63	32.3	4	22.2
**Parity**						
Nulliparous	74	34.1	67	34.5	7	38.9
Multiparous	138	65.1	127	65.5	11	61.1
**Number of fetuses**						
Singleton	205	96.2	190	97.4	15	83.3
Twins	8	3.8	5	2.6	3	16.7*
**Civil status**						
Single	12	6.1	11	6.2	1	5.6
Cohabiting	185	93.9	168	93.9	17	94.4
**Smoking**						
Yes	16	7.6	13	6.7	3	16.7
No	195	92.4	180	93.3	15	83.3
**BMI group**						
< 18.5	5	2.4	4	2.2	1	5.6
18.5–24.9	120	56.6	112	61.5	8	44.4
25.0–29.9	48	22.6	42	23.1	6	33.3
≥ 30.0	39	18.4	24	13.2	3	16.7
**Conception**						
Spontaneous	190	91.4	174	91.6	16	88.9
Assisted reproduction	18	8.7	16	8.4	2	11.1
**Ethnicity**						
Caucasian	197	92.5	180	92.3	17	94.4
Other	16	7.5	15	7.7	1	5.6
**Diagnosis abnormality**						
First trimester scan	133	62.4	128	65.6	5	27.8
Second trimester scan	80	37.6	67	34.4	13	72.2*
**Prognosis**						
Conditions with good long-term prognosis	13	6.1	9	4.6	4	22.2
Conditions with uncertain long-time prognosis	18	8.5	12	6.2	6	33.3
Conditions with poor long-term prognosis	67	31.5	60	30.8	7	38.9
Conditions with a lethal prognosis	13	6.1	13	6.7	0	0.0*
Conditions with a genetic disorder	102	47.9	101	51.8	1	5.6*

BMI: body mass index

^*^P < .001

Univariate regression analysis only showed an association between lethal condition and diagnosis in the first trimester (Table 4).

**Table 4:** Univariate and multivariate analysis for the association between demographic variables and the decision to terminate or continue the pregnancy

	Women informed about termination			Termination of pregnancy			
	Total	Cases	Cases	Univariate analysis		Multivariate analysis	
Variable	N	N	%	RR	(95% CI)	RR	(95% CI)
**Maternal age**							
18–24	24	22	91.7	1.04	(0.89—1.20)	N/A	N/A
25–29	61	54	88.5	1.00	(reference)	N/A	N/A
30–34	61	56	91.8	1.04	(0.92—1.17)	N/A	N/A
≥ 35	67	63	94.0	1.06	(0.95—1.18)	N/A	N/A
**Smoking status**							
No	195	180	92.3	1.00	(reference)	N/A	N/A
Yes	16	13	81.3	0.88	(0.69—1.12)	N/A	N/A
**Parity**							
Nulliparous	74	67	90.5	1.00	(reference)	N/A	N/A
Multiparous	138	127	92.0	1.02	(0.93—1.11)	N/A	N/A
**BMI group**							
< 18.5–24.99	125	116	92.8	1.00	(reference)	N/A	N/A
≥ 25.00	48	42	87.5	0.94	(0.84—1.06)	N/A	N/A
≥ 30.00	27	24	88.9	0.99	(0.90—1.10)	N/A	N/A
**Number of fetuses**							
Singleton	205	190	92.7	1.00	(reference)	N/A	N/A
Twins	8	5	62.5	0.67	(0.39—1.16)	N/A	N/A
**Civil status**							
Single	12	11	91.7	1.01	(0.85—1.20)	N/A	N/A
Cohabiting	185	168	90.8	1.00	(reference)	N/A	N/A
**Conception**							
Spontaneous	190	174	91.6	1.00	(reference)	N/A	N/A
Assisted reproduction	18	16	88.9	0.97	(0.82—1.15)	N/A	N/A
**Ethnicity**							
Caucasian	197	180	91.4	1.00	(reference)	N/A	N/A
Other	16	15	93.8	1.03	(0.90—1.17)	N/A	N/A
**Time of diagnosis**							
First trimester scan	133	128	96.2	1.00	(reference)	1.00	(reference)
Second trimester scan	80	67	83.6	0.87	(0.79—0.96)	0.91	(0.82—1.01)
**Prognosis malformations**							
Conditions with good long-term prognosis	13	9	69.2	1.00	(reference)	1.00	(reference)
Conditions with uncertain long-time prognosis	18	12	66.7	0.96	(0.59—1.57)	1.01	(0.63—1.62)
Conditions with poor long-term prognosis	67	60	89.6	1.29	(0.89—1.88)	1.36	(0.94—1.99)
Conditions with a lethal prognosis	13	13	100.0	1.44	(1.00—2.08)	1.45	(1.01—2.09)
Conditions with a genetic disorder	102	101	99.0	1.43	(0.99—2.06)	1.41	(0.98—2.03)

RR: risk ratio; CI: confidence interval; BMI: body mass index; N/A: not applicable

Multiple regression showed that a lethal prognosis was the only significant factor related to the parental decision to terminate the pregnancy when adjusting for the time of diagnosis (Table 4).

## Discussion

We found that information about termination of pregnancy was given in 67% of cases with a prenatally diagnosed malformation or genetic disorder. The proportion of parents who received information about termination increased with the severity of the long-term prognosis, from 12% for conditions with a good long-term prognosis to 100% for a lethal prognosis (Table 1). The distribution of abnormalities resulting in termination in our study is in line with other studies [4, 8, 15–18]. However, due to different abortion legislations among countries and due to the classification of the abnormalities, a comparison is difficult.

In conditions with a good long-term prognosis, it may seem surprising that 12% of the parents were informed about termination. A reason for this may be our classification of abnormalities. We speculate that the parents were informed about termination due to severe cases of diaphragmatic hernia, gastroschisis, or omphalocele because the prognosis was perceived to be poorer in these specific cases. Another reason may be diagnosis at early gestational age. Most of the abnormalities in this group were diagnosed at the FTS (77%). Furthermore, the residual risk of additional abnormalities that are undetectable by ultrasound or chromosomal microarray may have had an impact. The parents may also have requested information about termination [19, 20]. Some parents may request termination for conditions considered to have a good prognosis by obstetricians or specialists in fetal medicine. The parents may have experienced therapy failure in a sibling, perceived that the recommended therapy put a substantial burden on the family, or considered the condition to be more severe than the doctors did [4, 21]. In Denmark, doctors are legally bound to apply to the abortion council if parents wish to terminate the pregnancy, regardless of whether they agree. However, they do not themselves grant permission for termination, which is a significant difference between Denmark and other countries offering termination due to congenital abnormalities [4, 22, 23]. A study hypothesizes that this may protect the doctors in case of disagreement of the indication for termination [23]. The decision to inform parents about termination of pregnancy cannot be strictly made from a medical perspective because it also involves factors such as moral values, working conditions, psychological background and family circumstances among the parents, and possible health services [4].

In the category of conditions with a good long-term prognosis, 88% of the parents were not informed about termination (Table 1). Most of these cases included hydronephrosis, clubfoot, unilateral cystic renal dysplasia, and unilateral cleft lip. The chromosomal microarray was the standard prenatal genetic test during the study period and was performed prenatally in 34% of the malformation cases with normal test results. After birth, six of the 105 infants were diagnosed with an additional genetic disorder. This demonstrates that malformations with a good long-term prognosis may be associated with severe genetic disorders that are undetectable by microarray, changing the prognosis from good to poor. Today, it is possible to detect most of these genetic disorders prenatally by performing prenatalwhole exome/genome sequencing, but it is not a standard offer at the moment [24, 25]. In the uncertain long-term prognosis category, 69% of parents were informed about termination (Table 1). From an advisory perspective and a consideration of parental decision-making, counseling poses a greater challenge in this group because it is difficult to assess the prognosis. The prognosis may depend on the clinical manifestation of the malformation, which is not always possible to determine by ultrasound [21, 26]. Some cases may be associated with high neonatal mortality despite early treatment but have a good prognosis among the surviving infants [27]. In other cases, it may be possible to save the infant’s life, but with significant long-term morbidity and disability. One or more palliative procedures may provide good quality of life for many years. In these cases, it may be difficult for health professionals to provide clear and comprehensive information, leading to frustration and dissatisfaction among parents [26]. Furthermore, it may be difficult for parents to make an informed choice about whether to continue with the pregnancy based on complex probability estimates that are difficult to interpret objectively [28]. These challenges may be a reason for the varying information regarding termination in this group.

In line with other studies [4, 29, 30], all cases with a lethal prognosis, 98% of cases with a genetic disorder, and 96% of cases with a poor long-term prognosis were informed about termination (Table 1). Genetic disorder cases not informed about termination included Klinefelter and Turner syndrome. We classified all multiple malformation cases in the poor long-term prognosis category without regard to the prognosis of the malformations. In cases with less severe multiple malformations, the doctors may have considered the prognosis as good and therefore not informed the parents about termination.

A study suggests that screening policies have a significant influence on the practice of doctors [23]. The national screening policy in Denmark may be one reason for the uniform offer of termination in our study. However, personal perspectives and beliefs can be challenging to hide and are described as why different doctors make different decisions [22]. It may also lead to a power imbalance in the counseling process because it is the doctor who determines whether the indication is valid enough to justify an application for termination [5]. Parents may not know that doctors are duty bound to apply for pregnancy termination on their behalf if it is their wish regardless of indication. A doctor’s emphasis on an aspect of the matter can influence parental understanding of the condition and thereby influence the parental decision [28, 31].

Our study found that 92% of informed parents opted for termination (Table 2). It is a high fraction compared to other countries where termination of pregnancy for a fetal anomaly is legal [7, 30, 32]. We only included abnormalities detected before the fetus became viable, which may be a reason for the high percentage of parents opting for termination compared to other studies that included terminations after this limit. Gestational age is described to influence parental decision-making. Parents were less likely to choose termination with increasing gestational age [29, 33]. This could be explained by the severity of malformations detectable in the first trimester or due to more parental distress with advancing gestational age because of increased bonding. Termination early in pregnancy may also be less physically traumatizing for the mother [29, 34]. Our study showed the same tendency. Fewer parents decided to terminate the pregnancy if the abnormality was diagnosed in the second trimester.

We found that a lethal long-term prognosis was the only significant factor in the parental decision to choose termination (Table 4). The severity of abnormalities is also described to be the only consistent predictor for termination of the pregnancy in other studies [9, 10, 21, 32, 35].

Overall, cultural values and moral beliefs are described to play a significant role in parents’ attitudes to termination of pregnancy [11, 36, 37]. Parental considerations are reported to concern the affected infant, present and future siblings, career possibilities, increased domestic workload, and constant worrying [38]. Other studies found religion, education level, ethnicity, employment, age, income, obstetric history, and gestational age at diagnosis to influence the parental decision [9–11, 39–42]. The attitude of the Danish population toward selective termination is reported to be relatively liberal by international standards, except in the case of late termination for minor conditions [43, 44].

## Strengths and limitations

The unselected cohort, which included all hospitals in a region in Denmark with high participation in the screening program is a strength of this study [45, 46]. The usage of validated data from autopsy reports and postnatal medical records in a six month follow-up period after delivery is also a strength. There is a potential risk of misclassification of parental information in cases with a good long-term prognosis where medical records have no statement of information given, in contrast to terminated pregnancies where there is bound to be a legal document. We find that this potential misclassification will only underestimate the association between the severity of conditions and the parental decision to terminate a pregnancy.

The main limitation of this and other studies investigating factors relating to the decision to terminate a pregnancy is the classification of the abnormalities according to the long-term prognosis. In this and other studies, the authors classified abnormalities according to prognosis based on expert opinions, thus making it difficult to compare. We classified the validated abnormalities into five groups according to the long-term prognosis as this is described as being the main focus for the decision made by the parents [9–11]. We acknowledge that our classification may be subjective. To our knowledge, a validated classification of congenital abnormalities does not exist. Classification of the abnormalities was only based on ICD-10 diagnosis codes. Limited information on each abnormality may result in misclassification. Furthermore, our classification took into consideration that the abnormalities were diagnosed prenatally, which enabled immediate postnatal treatment in a highly specialized hospital, reducing mortality and morbidity in most of our cases. The prognosis of some abnormalities (e.g., diaphragmatic hernia) may be different if the infant does not receive highly specialized care immediately after birth.

This may explain why some parents were informed about termination while others were not for the same malformation. We cannot exclude the possible influence of the doctor’s personal opinion regarding the severity of the malformation. However, classification according to prognosis may be a strength compared to studies using the conditions themselves as an indication for termination of pregnancy.

The doctors usually document the information given to the parents, including information about the prognosis of the abnormality, possible treatments, and the possibility of applying for pregnancy termination if the condition meets the criteria of the abortion legislation. In most cases, the parental decision was documented in the medical records. Documentation was 100% in cases where the parents chose to terminate the pregnancy due to the mandatory legal documents completed for the abortion council.

In cases where information about pregnancy termination was not documented in the medical record, it was impossible to determine whether the parents did not receive the information or the doctor forgot to include documentation in the medical records. We believe it is less likely that doctors omit documentation regarding the information on pregnancy termination. In most cases, the doctor wrote that the parents were informed about the possibility of terminating the pregnancy, but the parents wished to continue.

By performing an observational cohort study based on a review of medical records, the nature of our study was limited to general variables of maternal demographic factors. An interview study could investigate the possible influence of religious conviction; the level of education; influence from family, friends, and society; general attitude toward abortion; and personal ethics, norms, and morality in a more nuanced way.

Furthermore, the small number in the continuation group affects the statistical strength of the study.

It has been speculated that the improved diagnostic possibilities in prenatal screening may shift the perception of severe conditions to less severe conditions and thereby trivialize termination [21]. Our data show that the most frequent indications for termination were lethal malformations and genetic anomalies. In the future, new treatment possibilities may be available and improve the prognosis for some conditions with a poor long-term prognosis. This may lead to fewer parents choosing termination. In recent decades, this shift has been seen with severe cardiac malformations, which went from being fatal conditions to having an expected survival rate of approximately 96% [47, 48].

Our study also shows the importance of detecting minor and less severe malformations, as they may be associated with severe genetic disorders. New methods in genetic testing, such as whole-exome/genome sequencing, may improve the accuracy of diagnosis and allow better prognostic information for clinical and parental decision-making.

## Conclusion

Our study showed that information about termination of pregnancy was mainly given for conditions with a poor or lethal long-term prognosis and genetic abnormalities. Only conditions with a lethal prognosis were significantly related to the parental decision to terminate the pregnancy.

## Supplementary Information

**Additional file 1: Box 1.** The Danish abortion legislation.

**Additional file 2: Box 2.** The Danish prenatal screening program.

**Additional file 3.** Malformations associated with genetic disorders.

## Data Availability

The authors do not wish to share the dataset. The datasets used and/or analyzed during the current study are not publicly available due to data protection regulations but are available from the corresponding author on reasonable request.

## Abbreviations

FTS: First-trimester scan
PAS: Patient administrative systems
REDCap: Research Electronic Data Capture
ICD-10: International Classification of Diseases and Related Health Problems
BMI: Body mass index
WHO: World Health Organization
OR: Odds ratio
RR: Risk ratio
aOR: Adjusted OR
CI: Confidence intervals
CNV: Copy number variation
