# Supplementary material for: Parental information about the option to apply for pregnancy termination after the detection of a congenital abnormality and factors influencing parental decision-making: a cohort study
Source: BMC Pregnancy Childbirth. 2022 Dec 17;22:948. doi: 10.1186/s12884-022-05255-0 (PMC9759856; doi:10.1186/s12884-022-05255-0)
Supplement: Supplementary file 1 — Additional file 1: Box 1. The Danish abortion legislation. [file 12884_2022_5255_MOESM1_ESM.docx]

**Box 1. The Danish abortion legislation**

The Danish abortion legislation allows free abortion up till 11+6 weeks of gestation.

From week 12+0, a regional abortion council must approve termination of pregnancy. The council consists of a gynecologist, a lawyer, and a psychologist/social worker.

Termination on fetal indication is permitted if there is a risk that the infant will be affected by:

- a severe physical or mental disability
- a hereditary condition
- an injury or disease during embryonic or fetal life

Permission for termination when the fetus is considered viable (after gestational age 22+6) is only granted in case of an exceptional severe condition.

In case of rejection by the regional abortion council, the woman has the right to apply to the appeals board.^1,2^
