# Supplementary material for: Parental information about the option to apply for pregnancy termination after the detection of a congenital abnormality and factors influencing parental decision-making: a cohort study
Source: BMC Pregnancy Childbirth. 2022 Dec 17;22:948. doi: 10.1186/s12884-022-05255-0 (PMC9759856; doi:10.1186/s12884-022-05255-0)
Supplement: Supplementary file 2 — Additional file 2: Box 2. The Danish prenatal screening program. [file 12884_2022_5255_MOESM2_ESM.docx]

**Box 2. The Danish prenatal screening program**

Since 2004, the Danish prenatal screening program are offered to all pregnant women free of charge and consists of two examinations.

The **first-trimester scan** can be performed when the fetal crown-rump length (CRL) is between 45mm and 84mm and includes an ultrasound examination to verify the fetus is alive, determine gestational age from the measurement of fetal crown-rump length, the number of fetuses, and an examination of the overall fetal anatomy. In addition, the pregnant woman can opt for a risk assessment for trisomy 13, 18, and 21, including measurement of the nuchal translucency and the result of the pregnancy-associated plasma protein-A (PAPP-A) and free beta-human chorionic gonadotrophin (beta-hCG) in the maternal blood.

Women with a risk ≥ 1:300 are offered invasive testing for genetic testing.^3^

The **second-trimester scan** is preferably performed between gestational weeks 19 and 21. The fetal anatomy is examined systematically for structural and developmental abnormalities in accordance with the Danish Fetal Medicine Society (DFMS) and the International Society of Ultrasound in Obstetrics and Gynecology (ISUOG) guidelines on routine measurements for the second-trimester scan.^4,5,6^

Both examinations are performed by sonographers. In case of abnormal findings, the parents are referred to a fetal medicine specialist or obstetrician for assessment.

The examinations. For the last ten years, the first-trimester and second-trimester scan uptake rate has been around 92% and 94%, respectively.^7^
