# Supplementary material for: Parental information about the option to apply for pregnancy termination after the detection of a congenital abnormality and factors influencing parental decision-making: a cohort study
Source: BMC Pregnancy Childbirth. 2022 Dec 17;22:948. doi: 10.1186/s12884-022-05255-0 (PMC9759856; doi:10.1186/s12884-022-05255-0)
Supplement: Supplementary file 3 — Additional file 3. Malformations associated with genetic disorders. [file 12884_2022_5255_MOESM3_ESM.docx]

| \| **Additional file 3** Malformations associated with genetic disorders \| \| \| \| \| \| --- \| --- \| --- \| --- \| --- \| \|  \| **Genetic disorder** \| \| \| \| \| **Malformations** \| Aneuploidy \| Sex chromosomal abnormality \| Monogenic disorders \| CNV \| \| **CNS** \|  \|  \|  \|  \| \| Holoprosencephaly \| <5 \|  \|  \|  \| \| Other cerebral hypoplasia \| <5 \|  \| <5 \|  \| \| Hydrocephalus \|  \|  \|  \| <5 \| \| **Face** \|  \|  \|  \|  \| \| Facial cleft \| <5 \| <5 \|  \|  \| \| Micrognathia \|  \|  \| <5 \|  \| \| **Thorax** \|  \|  \|  \|  \| \| Diaphragmatic hernia \| <5 \|  \|  \|  \| \| **Heart** \|  \|  \|  \|  \| \| Tetralogy of Fallot \|  \|  \|  \| <5 \| \| Hypoplastic left heart syndrome \| <5 \|  \|  \| <5 \| \| Ventricular septal defect \|  \|  \|  \| <5 \| \| Malformation of great arteries \|  \|  \|  \| <5 \| \| **Urinary tract** \|  \|  \|  \|  \| \| Hydronephrosis \|  \|  \|  \| <5 \| \| Megacystis/urethral valves \| <5 \|  \|  \|  \| \| **Abdominal wall** \|  \|  \|  \|  \| \| Omphalocele \| <5 \|  \|  \|  \| \| **Digestive system** \|  \|  \|  \|  \| \| Bowel atresia \| <5 \|  \|  \|  \| \| **Musculoskeletal** \|  \|  \|  \|  \| \| Clubfoot \|  \| 1 \| 1 \|  \| \| Shortened long bones \| <5 \|  \| 2 \|  \| \| Small biometries \|  \|  \| 1 \| 1 \| \| Congenital malformation of musculoskeletal system, unspecified \|  \|  \| <5 \|  \| \| **Other major fetal diseases** \|  \|  \|  \|  \| \| Hygroma \| <5 \|  \|  \|  \| \| Hydrops foetalis \| <5 \|  \|  \| <5 \| \| Tumor \|  \|  \|  \| <5 \| \| **Multiple malformations** \| <5 \|  \| <5 \| <5 \| \| Number is reported as < 5 to comply with data protection regulations \| \| \|  \|  \| |
| --- | --- | --- | --- | --- | --- | --- | --- | --- | --- | --- | --- | --- | --- | --- | --- | --- | --- | --- | --- | --- | --- | --- | --- | --- | --- | --- | --- | --- | --- | --- | --- | --- | --- | --- | --- | --- | --- | --- | --- | --- | --- | --- | --- | --- | --- | --- | --- | --- | --- | --- | --- | --- | --- | --- | --- | --- | --- | --- | --- | --- | --- | --- | --- | --- | --- | --- | --- | --- | --- | --- | --- | --- | --- | --- | --- | --- | --- | --- | --- | --- | --- | --- | --- | --- | --- | --- | --- | --- | --- | --- | --- | --- | --- | --- | --- | --- | --- | --- | --- | --- | --- | --- | --- | --- | --- | --- | --- | --- | --- | --- | --- | --- | --- | --- | --- | --- | --- | --- | --- | --- | --- | --- | --- | --- | --- | --- | --- | --- | --- | --- | --- | --- | --- | --- | --- | --- | --- | --- | --- | --- | --- | --- | --- | --- | --- | --- | --- | --- | --- | --- | --- | --- | --- | --- | --- | --- | --- | --- | --- | --- | --- | --- | --- | --- | --- | --- | --- | --- | --- | --- | --- | --- | --- | --- | --- |
